# Novel Mutations in *Titin* Exon 363 With Different Phenotypes Including a Founder Mutation in Eastern Europe

**DOI:** 10.1111/ene.70433

**Published:** 2025-11-17

**Authors:** Veronica Sian, Maria Francesca Di Feo, Sergei Kurbatov, Anna Vihola, Helena Luque, Fedor Konovalov, Stojan Peric, Cathrina Duffy, Cornelia Kornblum, Kristl G. Claeys, Peter Hackman, Bjarne Udd, Marco Savarese

**Affiliations:** ^1^ Folkhälsan Research Center Helsinki Finland; ^2^ Department of Medical and Clinical Genetics, Medicum University of Helsinki Helsinki Finland; ^3^ Department of Neuroscience, Rehabilitation, Ophthalmology, Genetics, and Maternal and Child Health (DINOGMI) University of Genoa Genoa Italy; ^4^ Research Institute of Experimental Biology and Medicine Voronezh State Medical University N.N. Burdenko Voronezh Russia; ^5^ Department of Neurology Named After K.N. Tretyakov Saratov State Medical University Named After V.I. Razumovsky Saratov Russia; ^6^ Laboratory of Genetics, HUS Diagnostic Center University of Helsinki and Helsinki University Hospital Helsinki Finland; ^7^ Independent Clinical Bioinformatics Laboratory Moscow Russia; ^8^ Neurology Clinic, University Clinical Center of Serbia, Faculty of Medicine University of Belgrade Belgrade Serbia; ^9^ Department of Neuromuscular Diseases, Center for Neurology University Hospital of Bonn Bonn Germany; ^10^ Department of Neurology University Hospitals Leuven Leuven Belgium; ^11^ Department of Neurosciences, Laboratory for Muscle Diseases and Neuropathies KU Leuven, and Leuven Brain Institute (LBI) Leuven Belgium; ^12^ Neuromuscular Research Center University of Tampere and Tampere University Hospital Tampere Finland

**Keywords:** congenital myopathy, founder mutation, M‐band, recessive distal myopathy, titin

## Abstract

**Background:**

Titin, the largest human protein, is essential for sarcomere structure and function. The *TTN* gene, spanning 364 exons, undergoes extensive alternative splicing thus producing multiple isoforms. The M‐band region, encoded by exons 359–364, plays a critical role in sarcomere integrity and mechanical stability. Exon 363 is of interest due to its involvement in titinopathies. Pathogenic truncating variants in this exon have been linked to recessive myopathies, including and mainly young‐onset recessive distal titinopathy.

**Methods:**

A multicenter study was conducted on six patients from five unrelated families with confirmed recessive titinopathy and truncating variants in exon 363. Clinical evaluations were performed. Genetic testing and segregation analysis confirmed the phase of the variants.

**Results:**

A novel truncating variant c.107578C>T, p.(Gln35860Ter) was identified in four unrelated patients of Eastern European ancestry, all carrying a second pathogenic variant in a canonical *TTN* exon. These patients exhibited juvenile/young‐adult onset recessive distal titinopathy with progressive lower limb weakness, frequently asymmetric muscle involvement, and no cardiac or respiratory complications. A Belgian family presented with a congenital myopathy caused by a novel frameshift deletion c.107430delA, p.(Ser35811AlafsTer32) in exon 363, in compound heterozygosity with a truncating variant in exon 208. These patients showed a more severe phenotype.

**Conclusions:**

This study expands the spectrum of TTN‐related myopathies, emphasizing exon 363's pathogenic significance. Truncating exon 363 variants contribute to young onset recessive distal and sometimes early onset titinopathy with contractures, and the phenotype severity is influenced by the second variant's location and exon usage.

## Graphical abstract

In six patients from five families, we identified two novel exon 363 pathogenic variants causing recessive titinopathies. Patients with a recurrent Eastern European founder variant presented with juvenile distal titinopathy, while a Belgian family showed an early‐onset titinopathy with contractures. Disease severity was influenced by the location of the second *TTN* variant.

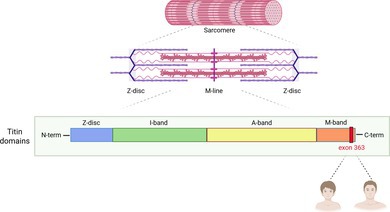

## 1 Introduction

Titin is the largest known protein in the human body, playing fundamental structural and functional roles in the sarcomere [1]. Encoded by the *TTN* gene, titin extends from the Z‐disk to the M‐band within the sarcomere [2, 3], acting as a molecular spring that contributes to muscle extensibility and mechanical stability [4]. The *TTN* gene consists of 363 coding exons and an additional first noncoding exon [5], and undergoes extensive alternative splicing [4]. The theoretical isoform incorporating all predicted *TTN* exons corresponds to the inferred full *TTN* “metatranscript” (NM_001267550.2) [4].

The C‐terminal, M‐band region of titin is encoded by the last 6 exons (359–364) [6]. M‐band titin consists of a serine/threonine kinase (TK) in the large M1 domain, followed by a series of alternating immunoglobulin domains (M1–M10) and unique insertion sequences (is1–is7) [6]. The second last *TTN* exon 363, which encodes the is7 domain in the M‐band, undergoes differential splicing, resulting in the is7− and is7+ isoforms [7, 8].

The M‐band plays a crucial role in the structural and mechanical stability of myofibrils, particularly regulating thick filament assembly [9]. It serves as a hub for protein–protein interactions, linking titin to myomesin, obscurin, and other key sarcomeric components [10].

Pathogenic variants in the M‐band segment of titin have been implicated in several muscle disorders with or without cardiomyopathies [4, 11]. Several truncating variants in exon 363 have been described so far, including a founder mutation in the Serbian population causing a juvenile onset recessive distal titinopathy [12, 13, 14, 15, 16]. Truncating variants in exon 363 result in a shorter titin and the lack of the C‐terminal portion most likely alters the mechanical and biochemical properties of the protein in muscle tissues [11, 17].

In the present study, we identified two novel nonsense variants in *TTN* exon 363. In a well‐defined cohort of patients with Eastern European ancestry, we identified a novel founder mutation, a nonsense variant c.107578C>T, p.(Gln35860Ter), causing young‐onset recessive distal titinopathy in trans with pathogenic variants in canonical exons. In a Belgian family, an early‐onset progressive Emery‐Dreifuss‐like titinopathy is caused by a previously unreported deletion c.107430delA, p.(Ser35811AlafsTer32) in exon 363 in compound heterozygosity with a single nucleotide deletion c.39524del, p.(Lys13175SerfsTer22) in exon 208.

## 2 Materials and Methods

### 2.1 Patients

All the patients or their legal guardians as well as the patient's relatives provided written informed consent to the referring clinician. The study, approved by the ethics committee of the Helsinki University Hospital (HUS/16896/2022), was performed in accordance with the Declaration of Helsinki.

In this multicenter study, we recruited six patients from five unrelated families with a confirmed recessive titinopathy and a truncating variant in exon 363. Electrophysiological examination results (nerve conduction studies and needle electromyogram (EMG)), creatine kinase (CK) measurements, histological and histochemical muscle examination results, and cardiac function test results were available for most patients (Table 1). Genetic testing was performed by a comprehensive gene panel and/or exome sequencing, and the phase of the variants was confirmed by segregation analysis. All variants are reported according to Human Genome Variation Society recommendations using the inferred complete *TTN* metatranscript as reference (NM_001267550.2). Exons are numbered 1–364 according to the LRG numbering as adopted by the Leiden Open Variation Database (LOVD, https://databases.lovd.nl/shared/genes/TTN).

**TABLE 1:** Summary of clinical data of six patients with distal myopathy.

ID	Sex	Age at onset	Age at last examination	Symptomps at onset	Independent walking	Weakness distribution	Cardiac involvement	Respiratory involvement	Muscle biopsy report	CK	EMG	Muscle MRI	Other	TTN var 1	Exon var 1	TTN var 2	Exon var 2
1	M	23	46	LL muscle wasting and muscle weakness	Yes	LL > UL	No	No	Rimmed vacuoles, fiber type variation, internal nuclei	Normal‐mildly elevated	NA	NA	Asymmetry	c.107578C>T p.(Gln35860Ter)	363	c.50228_50229del p.(Val16743AlafsTer19)	267—A‐band
2	F	26	58	LL muscle wasting and muscle weakness	Yes (steppage gait)	LL > UL	No	No	NA	Normal‐mildly elevated	Myogenic	Fatty degeneration of the right Adductor magnus and left semitendinosus, biceps femoris. Severe fatty replacement in soleus and anterior compartment muscles with gastrocnemius involvement on the left side	Asymmetry, thenar atrophy since the age of 50	c.107578C>T p.(Gln35860Ter)	363	c.66720C>G p.(Tyr22240Ter)	317—A‐band
3	M	20	37	Running difficulties	Yes	LL > UL	No	No	Autophagic vacuoles	Normal‐mildly elevated	Myogenic	Fatty degeneration of hamstrings and Adductor magnus and hypotrophy of left quadriceps on the thigh. Severe fatty replacement in soleus and tibialis anterior muscles	Asymmetry, rigid spin, reduced tendon reflexes	c.107578C>T p.(Gln35860Ter)	363	c.106571dup p.(Thr35525Aspfs*3)	361—M‐band
4	M	34	42	Walking difficulties	Yes	LL > UL	No	No	Prevalence of type 1 fiber, fiber splitting, increased central nuclei, vacuoles, degenerating, and regenerating fibers, a mild endomysial infiltrate (CD‐68) and mild inflammatory reaction	Mildly elevated	NA	Severe fatty changes in the anterolateral and soleus muscles of the lower legs	Calf pseudohypertrophy	c.107578C>T p.(Gln35860Ter)	363	c.107840T>A p.Ile35947Asn	364—M‐band
5_1	M	Childhood	51	Walking difficulties, contractures	No	LL > UL	No	Yes	Dystrophic features	Normal‐mildly elevated	Myogenic	No	NA	c.107431del p.(Ser35811AlafsTer33)	363	c.39524del p.(Lys13175SerfsTer22)	208—A‐band
5_2	M	Childhood	48	Walking difficulties, contractures	No	LL > UL	No	Yes	NA	Normal‐mildly elevated	NA	No	NA	c.107431del p.(Ser35811AlafsTer33)	363	c.39524del p.(Lys13175SerfsTer22)	208—A‐band

*Note:* All variants correspond to transcript NM_001267550.2.

Abbreviations: CK, creatine kinase; EMG, needle electromyogram; F, female; LL, lower limb; M, male; MRI, muscle magnetic resonance image; NA, data not available; UL, upper limb.

Exon usage (expressed as PSI, percentage spliced in) has been retrieved by an online resource (gacatag.shinyapps.io/TTN_PSIVIS/) and calculated as reported previously [18].

### 2.2 Western Blotting

Frozen muscle biopsies were used to prepare Western blot samples as described before [11, 19]. Samples were run on SDS–PAGE gels (12%) using Bio‐Rad Mini‐Protean equipment (Bio‐Rad, Hercules, CA), and transferred onto polyvinylidene difluoride (PVDF) membranes. Two previously described in‐house–generated antibodies (rabbit polyclonal antibody M10‐1 [11] and mouse monoclonal antibody 11‐4‐3 [19]) were used to detect the titin via the M10 domain, followed by horseradish peroxidase–conjugated secondary antibodies (Dako) and enhanced chemiluminescent detection using the Pierce SuperSignal West Femto substrate (Thermo Fisher).

## 3 Results

### 3.1 Juvenile‐Young Adult Onset Recessive Distal Titinopathy

Targeted next‐generation sequencing of myopathy‐related genes identified a novel truncating variant c.107578C>T, p.(Gln35860Ter) in four unrelated patients with a juvenile–young adult‐onset distal myopathy. The four patients, one is from Serbia, two patients are from Russia and the fourth one from Germany, share the same causative variant. All the patients carry a second pathogenic variant in a canonical *TTN* exon, respectively c.50228_50229del p.(Val16743AlafsTer19) in exon 267 (A‐band), c.66720C>G p.(Tyr22240Ter) in exon 317 (A‐band), c.106571dup p.(Thr35525AspfsTer3) in exon 361 (M‐band), and previously reported missense c.107840T>A p.(Ile35947Asn) in exon 364 (M‐band). The same *TTN* haplotype was observed for patients 1, 2 and 4, for whom raw genetic data was available (Table 2).

**TABLE 2:** *TTN* haplotype.

Position (hg19)	ExonicFunc	AAChange	Exon
179392275	stopgain	c.107578C>T:p.Q35860X	363
179424048	synonymous_SNV	c.86811A>G:p.V28937V	327
179498042	nonsynonymous_SNV	c.42958A>G:p.K14320E	234
179583317	synonymous_SNV	c.24516C>T:p.T8172T	86
179586604	nonsynonymous_SNV	c.22786G>C:p.D7596H	79
179638238	synonymous_SNV	c.7545C>T:p.Y2515Y	32

All the patients have been on regular follow‐up for many years, with a minimum of 8 years in Patient 4 and a maximum of 32 years in Patient 2. In all cases, a slowly progressive course was reported. The average age at the last evaluation was 46 years (range 37–58), and all patients were still ambulant.

Patient 1 showed the first symptoms at the age of 23 years. He developed right limb's muscle atrophy, with muscle weakness more pronounced in the lower limbs than in the upper limbs. Prominent asymmetric muscle weakness and atrophy were observed in the right part of the body. Muscle biopsy (no longer available) evidenced myopathic changes with rimmed vacuoles, increased fiber type variation, and internal nuclei. At last examination, at the age of 46, the patient was still able to walk independently, and cardiac and respiratory involvement were ruled out.

**FIGURE 1:**
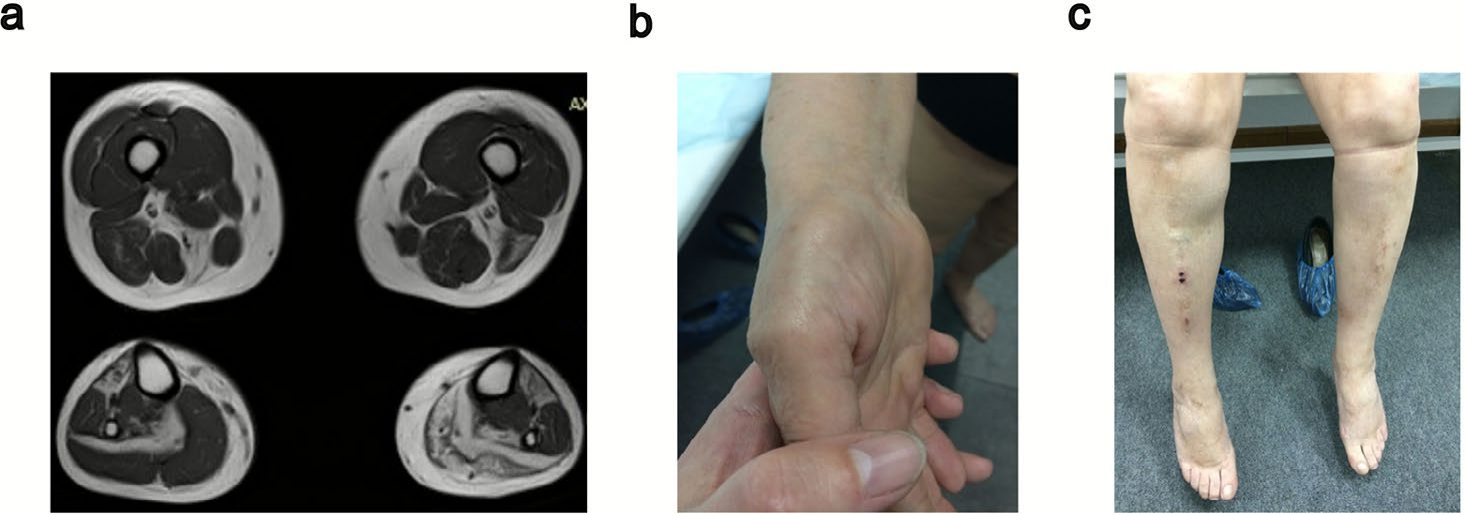
Main clinical findings in Patient 2. (a) Muscle MRI of the lower limbs showing less severe changes in the thigh with fatty degeneration on the right in Adductor magnus and semitendinosus, biceps femoris on the left side, and the typical early changes observed in juvenile onset recessive distal titinopathy with severe fatty replacement in soleus and anterior compartment muscles (in this case, also asymmetric gastrocnemius involvement on the left side). (b) Thenar and mild hypothenar hypotrophy. (c) Distal lower leg weakness.

Patient 2 showed muscle wasting in the lower leg with consequent muscle weakness at the age of 26 years. She used to be an active person and walk long distances until her twenties. Fatigue and weakness in the lower legs (more on one side), worsened over time and steppage gait has been present from the age of 28. Mild weakness of neck flexors was also noticed, and CK levels were mildly elevated (4×ULN). EMG showed myopathic anomalies, with no spontaneous activity, and MRI at the age of 58 displayed asymmetric muscle atrophy, with diffuse fatty replacement in the left leg, and mostly in the anterior part of the right leg (Figure 1a). Thenar hypotrophy and mild hypothenar hypotrophy (left > right) were also evident (Figure 1b), together with lower leg involvement with both peroneal and tibial weakness (Figure 1c). No respiratory or cardiac involvement was reported at the age of 58.

**FIGURE 2:**
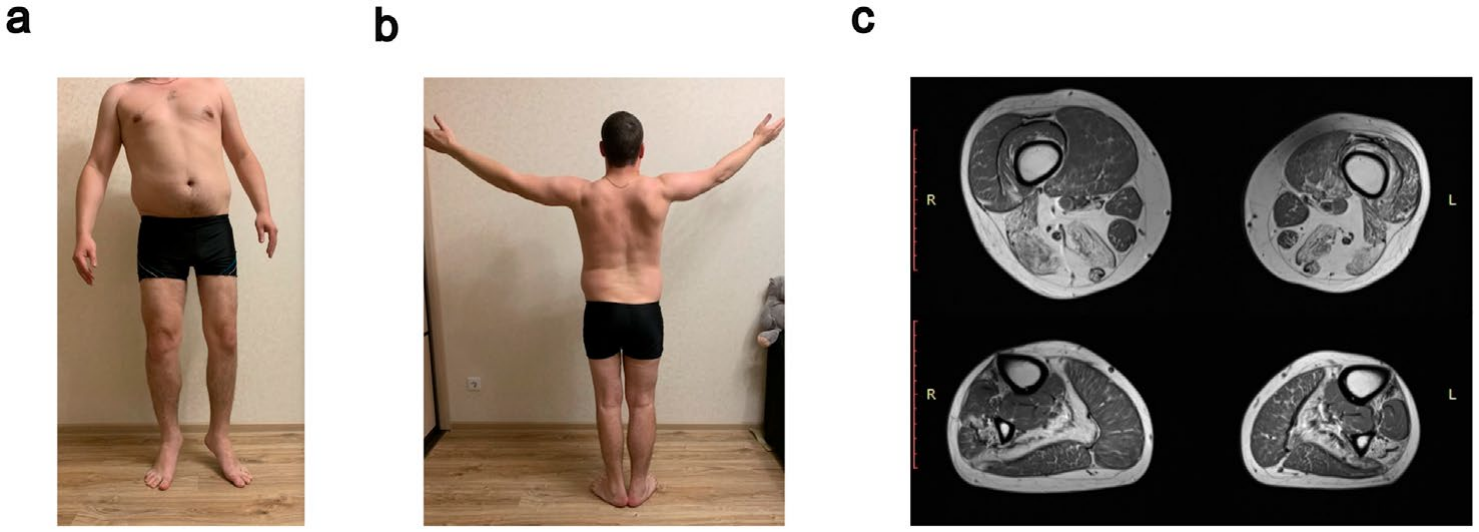
Main clinical findings in Patient 3. (a) Asymmetric lower limb atrophy, left > right. (b) Mild asymmetric scapular winging. (c) Muscle MRI of the lower limbs showing fatty degeneration of hamstrings and adductor magnus and hypotrophy of the left quadriceps on the thigh. And the typical changes in juvenile onset recessive distal titinopathy with severe fatty replacement in soleus and tibialis anterior muscles (in this case, also asymmetric peroneus involvement).

Patient 3 had an early onset at the age of 20 years, reporting slight weakness in his left leg while running. At the last examination, at the age of 37, he showed clear asymmetrical atrophy of the lower limbs, with more pronounced involvement of the left leg (Figure 2a), and mild asymmetrical scapular winging (Figure 2b). Rigid spine and reduced tendon reflexes were also observed. Cardiac ultrasound and forced vital capacity were normal. CK values showed fluctuations over the years (between 4×ULN and 6×ULN), without a clear correlation with the clinical symptoms. At the age of 30, muscle MRI showed involvement of the tibial anterior, soleus and peroneal muscles in the distal lower limbs, and hamstrings, vastus intermedius and vastus medialis in the thigh (Figure 2c).

**FIGURE 3:**
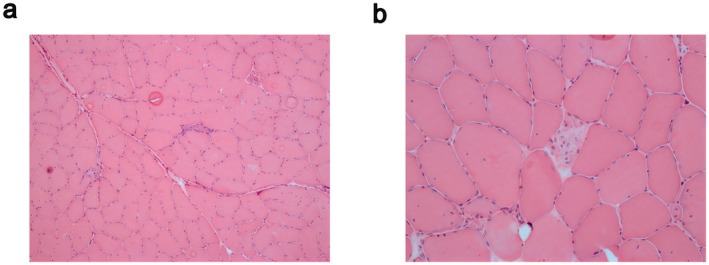
Histologic examination of muscle biopsy. Hematoxylin and eosin (H&E) staining of muscle biopsy from left gastrocnemius of Patient 4. Magnification: (a) 10×, (b) 20×.

**FIGURE 4:**
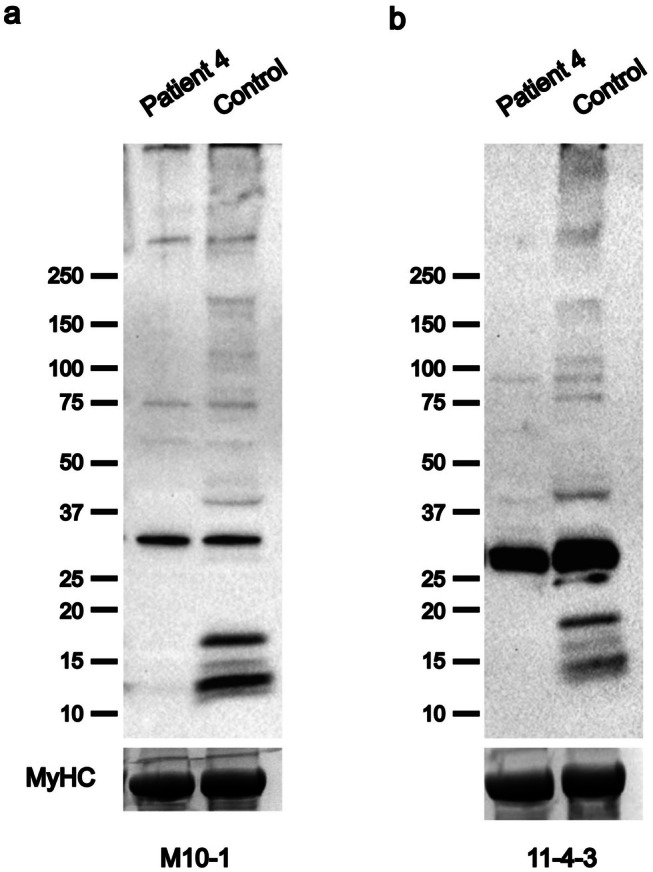
Western blot for C‐Terminal Titin Fragments. Western blotting using two different antibodies, (a) M10‐1 and (b) 11‐4‐3, against the titin C‐terminal M10 domain. Patient 4 with a single identified protein truncating variant shows a severe reduction of titin C‐terminal fractions. The myosin heavy chain (MyHC) serves as the loading control.

Patient 4 reported, at age 34, difficulties in walking with muscle weakness predominant in the lower limbs. Absence of cardiac and respiratory involvement was observed in the patient at age 42 years. Muscle MRI revealed severe fatty changes in the anterolateral and soleus muscles of the lower legs. The hamstring muscles were the main severely affected muscles of the thighs, and gluteus medius and minimus were the most affected in the pelvic. Muscle biopsy performed at the age of 37 from the left gastrocnemius showed predominance of type 1 fibers, fiber splitting, increased central nuclei, vacuoles, some degenerating and regenerating fibers, a mild endomysial infiltrate (CD‐68) and mild inflammatory reaction without clear signs of myositis. Hematoxylin and Eosin (H&E) staining (Figure 3a,b) evidenced fiber size variation, fibers with increased central nuclei, few atrophic fibers as well as degenerating and regenerating fibers, vacuoles and fiber splitting. His father, carrier of the Belgian Tibial Muscular Dystrophy (TMD)‐causing variant c.107840T>A p.(Ile35947Asn) in exon 364 [20], showed bilateral foot drop at the age of 75, with unsteady and steppage gait. Western blotting analyses showed a marked reduction of small C‐terminal titin protein fragments, indicating a decrease in their expression (Figure 4a,b).

### 3.2 Early Onset Progressive Titinopathy With Contractures

Patients 5a and 5b, two affected brothers with an early onset, were found compound heterozygous for a frameshift variant c.107431del p.(Ser35811AlafsTer33) in exon 363 and a second single nucleotide deletion c.39524del p.(Lys13175SerfsTer22) in exon 208 (A‐band).

They both presented with contractures, proximal weakness, and atrophy as with Emery‐Dreifuss‐like phenotype without cardiomyopathy. One brother had a more severe clinical presentation leading to wheelchair confinement since adolescence (currently 51 years), while the other was wheelchair bound since age 25 years (currently 48 years). Cardiac examinations were normal. Lung function tests at the current age of 48 and 51 years showed restrictive lung disease, with FVC 68% (significantly reduced below 80% since age 46 years) and 31% (unknown since what age significantly reduced; no NIV), respectively.

## 4 Discussion

**FIGURE 5:**
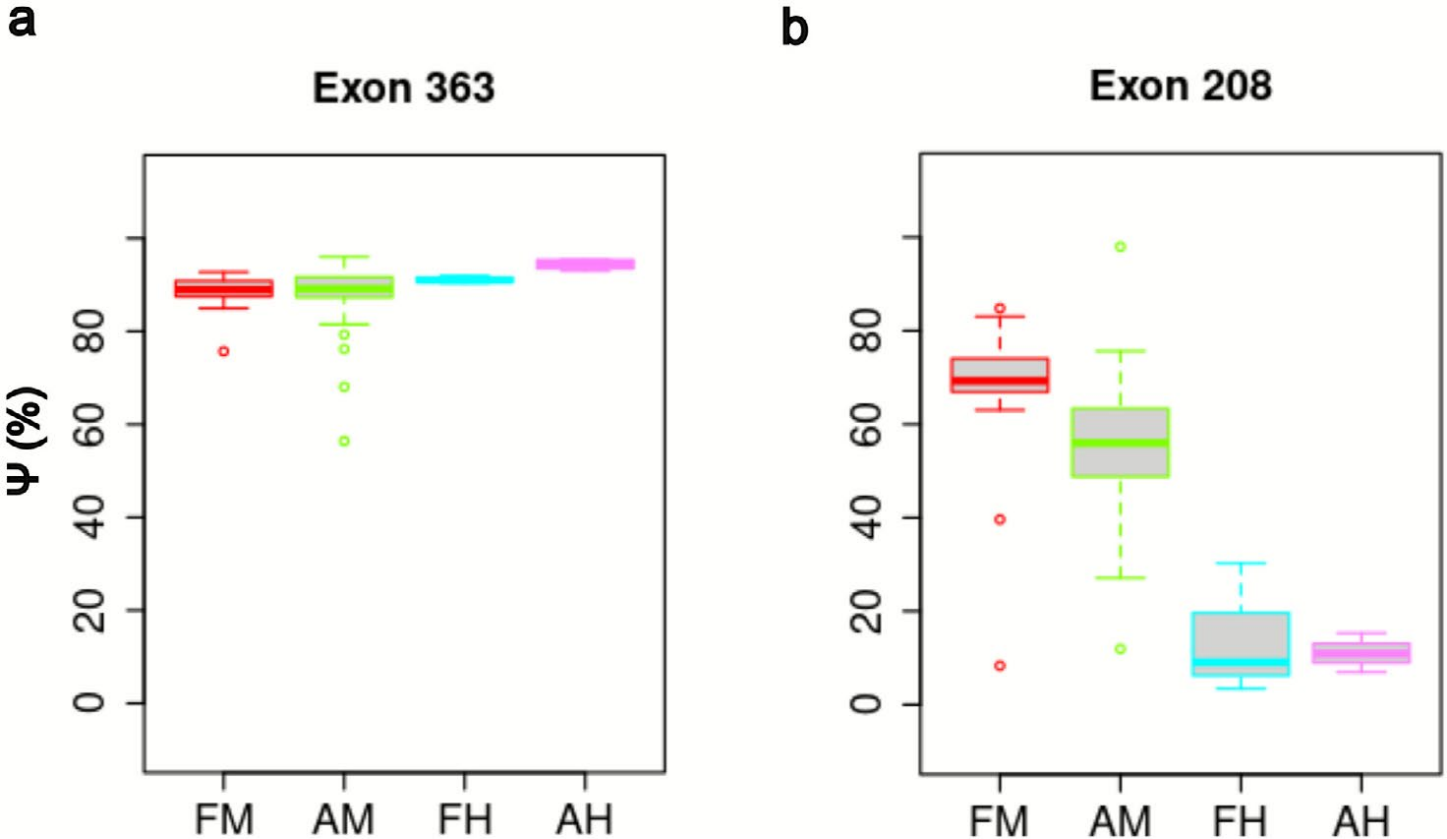
Exon usage of *TTN*. PSI box plot representation of (a) exon 363 and (b) exon 208 of *TTN* in prenatal (red) and postnatal (green) skeletal muscles. AH, postnatal heart; AM, postnatal muscle; FH, fetal heart; FM, fetal muscle.

Among the many exons that encode Titin, exon 363 has drawn particular interest due to its localization within the M‐band and its role in modulating Titin's structural properties [11]. Exon 363 encodes a segment of the Titin protein that is crucial for sarcomere integrity and contributes to the functional specialization of different muscle types [21, 22]. In addition, the inclusion or exclusion of exon 363 through alternative splicing results in distinct Titin isoforms may influence muscle mechanical resilience (Figure 5a) [18, 23].

Despite being an alternatively spliced exon, pathogenic variants affecting exon 363 have been identified in many titinopathy patients [13]. A common founder mutation c.107635C>T p.(Gln35879Ter) has been identified in patients of Serbian origin showing a recessive distal myopathy with a juvenile/early‐adult onset [15]. Similarly, we here report a novel founder mutation c.107578C>T, p.(Gln35860Ter), once again in the Eastern European population, causing a similar distal titinopathy. The variant has a frequency of 0.002338% in the gnomAD European non‐Finnish population and it has been observed in a single allele in the patient subgroup of a Russian database (http://ruseq.ru, total number of alleles = 9550), a large‐scale reference database of genetic variants specific to the Russian population [24].

In the first three patients, the combination of the novel founder mutation and truncating variants in canonical exons results in a similar phenotype due to the complete lack of a titin protein expressing the entire M9 as demonstrated by the strong reduction/absence of C‐terminal fragments in the Western blot in Patient 4 (Figure 4a,b). In addition to the exon 363 variant, Patient 4 has a previously reported pathogenic missense variant in exon 364 p.(Ile35947Asn) [20]. The missense variant in the last exon causes a reduction in C‐terminal fragments due to aberrant proteolytic processing of the titin C‐terminus [16]. Interestingly, Patients 5a and 5b show a more severe phenotype with an early onset progressive titinopathy with contractures, and wheelchair confinement already in adolescence/early adulthood. Although we still lack a clear genotype–phenotype correlation, other congenital/infantile cases with a truncating variant in the last two exons have been reported [19, 25]. The specific location of the second truncating variant and the PSI of the mutated exon explain the phenotype. In patients 1–4, the second variants are located in different exons that have a PSI comparable to that of exon 363 (i.e., over 80% in fetal and postnatal skeletal muscles) (Table S1). On the contrary, in family 5, the second variant is located in exon 208 showing a PSI of 67% in fetal muscles and 56% in postnatal muscles (Figure 5b).

In conclusion, our study confirms that a recessive distal titinopathy with young or early‐adult onset occurs from either two truncating mutations in the final two exons (363–364) or a combination of one mutation in these exons and a truncating mutation in a canonical exon on the other allele. In line with previous studies, our cohort also confirms the predominant lower limb involvement, the presence of a normal to mildly elevated serum creatine kinase level, and, above all, the lack of cardiac and respiratory involvement. Interestingly, all the patients we report here show marked asymmetric muscle involvement, that has been similarly observed in 5/14 patients with the Serbian founder mutation.

However, our study also confirms that truncating variants in exon 363 may also result in early‐onset myopathies in the presence of a second variant in exons with specific exon usage.

## Author Contributions

**Veronica Sian:** conceptualization, data curation, writing‐original draft, writing – review and editing. **Maria Francesca Di Feo:** conceptualization, data curation, writing – review and editing. **Sergei Kurbatov:** investigation, resources, writing – review and editing. **Anna Vihola:** investigation, writing – review and editing. **Helena Luque:** investigation, writing – review and editing. **Fedor Konovalov:** investigation, resources, writing – review and editing. **Stojan Peric:** investigation, resources, writing – review and editing. **Cathrina Duffy:** investigation, resources, writing – review and editing. **Cornelia Kornblum:** investigation, resources, writing – review and editing. **Kristl G. Claeys:** investigation, resources, writing – review and editing. **Peter Hackman:** conceptualization, writing – review and editing. **Bjarne Udd:** conceptualization, investigation, supervision, writing – review and editing. **Marco Savarese:** conceptualization, data curation, supervision, writing‐original draft, writing – review and editing.

## Conflicts of Interest

The authors declare no conflicts of interest.

## Supporting information


**Table S1:** Exon usage, expressed as percentage spliced in (PSI), of each mutated exon in fetal and adult muscles.

## Data Availability

The data that support the findings of this study are available from the corresponding author upon reasonable request.

## References

[1] M. Krüger and S. Kötter , “Titin, a Central Mediator for Hypertrophic Signaling, Exercise‐Induced Mechanosignaling and Skeletal Muscle Remodeling,” Frontiers in Physiology 7 (2016): 76, 10.3389/fphys.2016.00076. 26973541 PMC4771757

[2] S. Labeit and B. Kolmerer , “Titins: Giant Proteins in Charge of Muscle Ultrastructure and Elasticity,” Science 270, no. 5234 (1995): 293–296, 10.1126/science.270.5234.293. 7569978

[3] D. O. Fürst , M. Osborn , R. Nave , and K. Weber , “The Organization of Titin Filaments in the Half‐Sarcomere Revealed by Monoclonal Antibodies in Immunoelectron Microscopy: A Map of Ten Nonrepetitive Epitopes Starting at the Z Line Extends Close to the M Line,” Journal of Cell Biology 106, no. 5 (1988): 1563–1572, 10.1083/jcb.106.5.1563. 2453516 PMC2115059

[4] M. Savarese , J. Sarparanta , A. Vihola , B. Udd , and P. Hackman , “Increasing Role of Titin Mutations in Neuromuscular Disorders,” Journal of Neuromuscular Diseases 3, no. 3 (2016): 293–308, 10.3233/JND-160158. 27854229 PMC5123623

[5] M. L. Bang , T. Centner , F. Fornoff , et al., “The Complete Gene Sequence of Titin, Expression of an Unusual ≈700‐kDa Titin Isoform, and Its Interaction With Obscurin Identify a Novel Z‐Line to I‐Band Linking System,” Circulation Research 89, no. 11 (2001): 1065–1072, 10.1161/hh2301.100981. 11717165

[6] A. Biquand , S. Spinozzi , P. Tonino , et al., “Titin M‐Line Insertion Sequence 7 Is Required for Proper Cardiac Function in Mice,” Journal of Cell Science 134, no. 18 (2021): jcs258684, 10.1242/jcs.258684. 34401916 PMC8466004

[7] B. Kolmerer , N. Olivieri , C. C. Witt , B. G. Herrmann , and S. Labeit , “Genomic Organization of M Line Titin and Its Tissue‐Specific Expression in Two Distinct Isoforms,” Journal of Molecular Biology 256, no. 3 (1996): 556–563, 10.1006/jmbi.1996.0108. 8604138

[8] K. Charton , L. Suel , S. F. Henriques , et al., “Exploiting the CRISPR/Cas9 System to Study Alternative Splicing In Vivo: Application to Titin,” Human Molecular Genetics 25, no. 20 (2016): 4518–4532, 10.1093/hmg/ddw280. 28173117

[9] Z. Chen , R. Maimaiti , C. Zhu , et al., “Z‐Band and M‐Band Titin Splicing and Regulation by RNA Binding Motif 20 in Striated Muscles,” Journal of Cellular Biochemistry 119, no. 12 (2018): 9986–9996, 10.1002/jcb.27328. 30133019 PMC6218289

[10] A. Fukuzawa , S. Lange , M. Holt , et al., “Interactions With Titin and Myomesin Target Obscurin and Obscurin‐Like 1 to the M‐Band—Implications for Hereditary Myopathies,” Journal of Cell Science 121, no. 11 (2008): 1841–1851, 10.1242/jcs.028019. 18477606

[11] P. Hackman , S. Marchand , J. Sarparanta , et al., “Truncating Mutations in C‐Terminal Titin May Cause More Severe Tibial Muscular Dystrophy (TMD),” Neuromuscular Disorders 18, no. 12 (2008): 922–928, 10.1016/j.nmd.2008.07.010. 18948003

[12] J. Sarparanta , G. Blandin , K. Charton , et al., “Interactions With M‐Band Titin and Calpain 3 Link Myospryn (CMYA5) to Tibial and Limb‐Girdle Muscular Dystrophies,” Journal of Biological Chemistry 285, no. 39 (2010): 30304–30315, 10.1074/jbc.M110.108720. 20634290 PMC2943315

[13] P. Hackman , A. Vihola , H. Haravuori , et al., “Tibial Muscular Dystrophy Is a Titinopathy Caused by Mutations in TTN, the Gene Encoding the Giant Skeletal‐Muscle Protein Titin,” American Journal of Human Genetics 71 (2002): 492–500. 12145747 10.1086/342380 PMC379188

[14] B. Udd , A. Vihola , J. Sarparanta , I. Richard , and P. Hackman , “Titinopathies and Extension of the M‐Line Mutation Phenotype Beyond Distal Myopathy and LGMD2J,” Neurology 64, no. 4 (2005): 636–642, 10.1212/01.WNL.0000151853.50144.82. 15728284

[15] S. Perić , J. N. Glumac , A. Töpf , et al., “A Novel Recessive TTN Founder Variant Is a Common Cause of Distal Myopathy in the Serbian Population,” European Journal of Human Genetics 25, no. 5 (2017): 572–581, 10.1038/ejhg.2017.16. 28295036 PMC5437897

[16] A. Evilä , J. Palmio , A. Vihola , et al., “Targeted Next‐Generation Sequencing Reveals Novel TTN Mutations Causing Recessive Distal Titinopathy,” Molecular Neurobiology 54, no. 9 (2017): 7212–7223, 10.1007/s12035-016-0242-3. 27796757

[17] M. Savarese , A. Vihola , E. C. Oates , et al., “Genotype–Phenotype Correlations in Recessive Titinopathies,” Genetics in Medicine 22, no. 12 (2020): 2029–2040, 10.1038/s41436-020-0914-2. 32778822

[18] M. F. Di Feo , A. Oghabian , E. Nippala , et al., “Inferring Disease Course From Differential Exon Usage in the Wide Titinopathy Spectrum,” Annals of Clinical and Translational Neurology 1 (2024): 2745–2755, 10.1002/acn3.52189. PMC11514934 39198997

[19] A. Evilä , A. Vihola , J. Sarparanta , et al., “Atypical Phenotypes in Titinopathies Explained by Second Titin Mutations,” Annals of Neurology 75, no. 2 (2014): 230–240, 10.1002/ana.24102. 24395473

[20] P. Y. K. Van den Bergh , O. Bouquiaux , C. Verellen , et al., “Tibial Muscular Dystrophy in a Belgian Family,” Annals of Neurology 54, no. 2 (2003): 248–251, 10.1002/ana.10647. 12891679

[21] S. Lange , N. Pinotsis , I. Agarkova , and E. Ehler , “The M‐Band: The Underestimated Part of the Sarcomere,” Biochimica et Biophysica Acta 1867, no. 3 (2020): 118440, 10.1016/j.bbamcr.2019.02.003. 30738787 PMC7023976

[22] D. Stroik , Z. R. Gregorich , F. Raza , Y. Ge , and W. Guo , “Titin: Roles in Cardiac Function and Diseases,” Frontiers in Physiology 15 (2024): 15, 10.3389/fphys.2024.1385821. PMC11040099 38660537

[23] M. Savarese , P. H. Jonson , S. Huovinen , et al., “The Complexity of Titin Splicing Pattern in Human Adult Skeletal Muscles,” Skeletal Muscle 8, no. 1 (2018): 11, 10.1186/s13395-018-0156-z. 29598826 PMC5874998

[24] Y. A. Barbitoff , D. N. Khmelkova , E. A. Pomerantseva , et al., “Expanding the Russian Allele Frequency Reference via Cross‐Laboratory Data Integration: Insights From 7,452 Exome Samples,” *medRxiv*, Published online January 1, 2022:2021.11.02.21265801, 10.1101/2021.11.02.21265801. PMC11533896 39498263

[25] E. Harris , A. Töpf , A. Vihola , et al., “A ‘Second Truncation’ in TTN Causes Early Onset Recessive Muscular Dystrophy,” Neuromuscular Disorders 27, no. 11 (2017): 1009–1017, 10.1016/j.nmd.2017.06.013. 28716623

